# Studies Involving People With Dementia and Touchscreen Technology: A Literature Review

**DOI:** 10.2196/rehab.5788

**Published:** 2016-11-04

**Authors:** Phil Joddrell, Arlene J Astell

**Affiliations:** ^1^Centre for Assistive Technology and Connected Healthcare (CATCH)School of Health and Related Research (ScHARR)University of SheffieldSheffieldUnited Kingdom

**Keywords:** dementia, technology, literature review

## Abstract

**Background:**

Devices using touchscreen interfaces such as tablets and smartphones have been highlighted as potentially suitable for people with dementia due to their intuitive and simple control method. This population experience a lack of meaningful, engaging activities, yet the potential use of the touchscreen format to address this issue has not been fully realized.

**Objective:**

To identify and synthesize the existing body of literature involving the use of touchscreen technology and people with dementia in order to guide future research in this area.

**Methods:**

A systematized review of studies in the English language was conducted, where a touchscreen interface was used with human participants with dementia.

**Results:**

A total of 45 articles met the inclusion criteria. Four questions were addressed concerning (1) the context of use, (2) reasons behind the selection of the technology, (3) details of the hardware and software, and (4) whether independent use by people with dementia was evidenced.

**Conclusions:**

This review presents an emerging body of evidence demonstrating that people with dementia are able to independently use touchscreen technology. The intuitive control method and adaptability of modern devices has driven the selection of this technology in studies. However, its primary use to date has been as a method to deliver assessments and screening tests or to provide an assistive function or cognitive rehabilitation. Building on the finding that people with dementia are able to use touchscreen technology and which design features facilitate this, more use could be made to deliver independent activities for meaningful occupation, entertainment, and fun.

## Introduction

Dementia is an incurable syndrome caused by a chronic or progressive disease of the brain [1]. It has currently affected more than 46 million people worldwide, and this number is predicted to increase to 131.5 million by 2050 [2]. Dementia can affect multiple areas of cognitive functioning, including memory, thinking, comprehension, learning capacity, orientation, judgment, and language, and many people experience an impact on motivation, social behavior and emotion [1].

Lack of activity, or boredom, is frequently reported by people with dementia, whether they are still living at home or have moved into care services [3,4]. Engaging in meaningful activities can decrease boredom and increase positive emotions [5]. Facilitating people with dementia to engage in independent activity through the selection of appropriate activities can be highly beneficial as it promotes autonomy, thereby avoiding dependence on family members or formal caregivers [6].

The use of technology in dementia care is growing [7], but it has been observed that technological solutions developed for people with dementia have been centered around “assistive” devices [8-10]. Ironically, these applications are typically not intended for use by the people with dementia, but rather by family members or formal caregivers [11]. Furthermore, there has been some debate surrounding the use of technological assistance in this context, particularly in cases involving the monitoring or control of individuals through “assistive” devices, such as electronic tagging [8]. This highlights the need for careful consideration when introducing technological devices as aids for people with dementia, and to be clear from the outset who the “assistance” is actually for.

The increased availability of touchscreen technology devices in everyday life, such as smartphones and tablets, has led to an increased consideration by health care professionals and researchers of their potential suitability for people with dementia [12]. This trend is set to continue as people are being diagnosed with dementia at a younger age, and coming generations will be more familiar with computer technology [13]. It has been suggested that the touchscreen format is a more effective solution as it makes less demand of hand-eye coordination when compared with a desktop computer using a mouse and cursor [14]. Therefore, the intuitive nature of touchscreen devices presents an opportunity for their application with people with dementia as the intended users of the technology, and for whom the benefits may be experienced directly. For this potential to be realized, the design of simple and accessible software should be considered a priority.

This review presents an overview of the ways touchscreen technology has been used with people with dementia since its invention to the present generation of touchscreen devices, addressing the questions listed in Textbox 1.

**Questions addressed by the literature review.**

- In which contexts has touchscreen technology been used by people with dementia?
- For what reason was touchscreen technology chosen?
- Which forms of hardware and software were used?
- Is there any evidence that people with dementia were able to use touchscreen technology independently?

## Methods

A systematized review [15] of the literature was conducted on the use of touchscreen technology with people with dementia.

The following search terms, including Boolean operators (eg, AND, OR) and truncation symbols (denoted by *), were used for this review: (dementia) OR (Alzheimer*) AND (touchscreen) OR (touch screen) OR (tablet computer) OR (tablet device) OR (smartphone) OR (smart phone) AND (app*) OR (activit*) OR (game*) OR (gaming).

The following electronic databases were accessed for this review, selected due to their content being relevant to the subject area: Medline via Web of Science; PsychINFO via Ovid SP; ProQuest; PubMed; CINAHL via EBSCO; and Cochrane. The search was extended to include references of relevant articles and existing articles in the researcher’s reference management database. The literature search was conducted between July 20 and August 7, 2015.

During screening, records were included or excluded based on the following criteria: Language: English, Participants: human with dementia, and Technology: any featuring a touchscreen interface.

**Figure 1:**
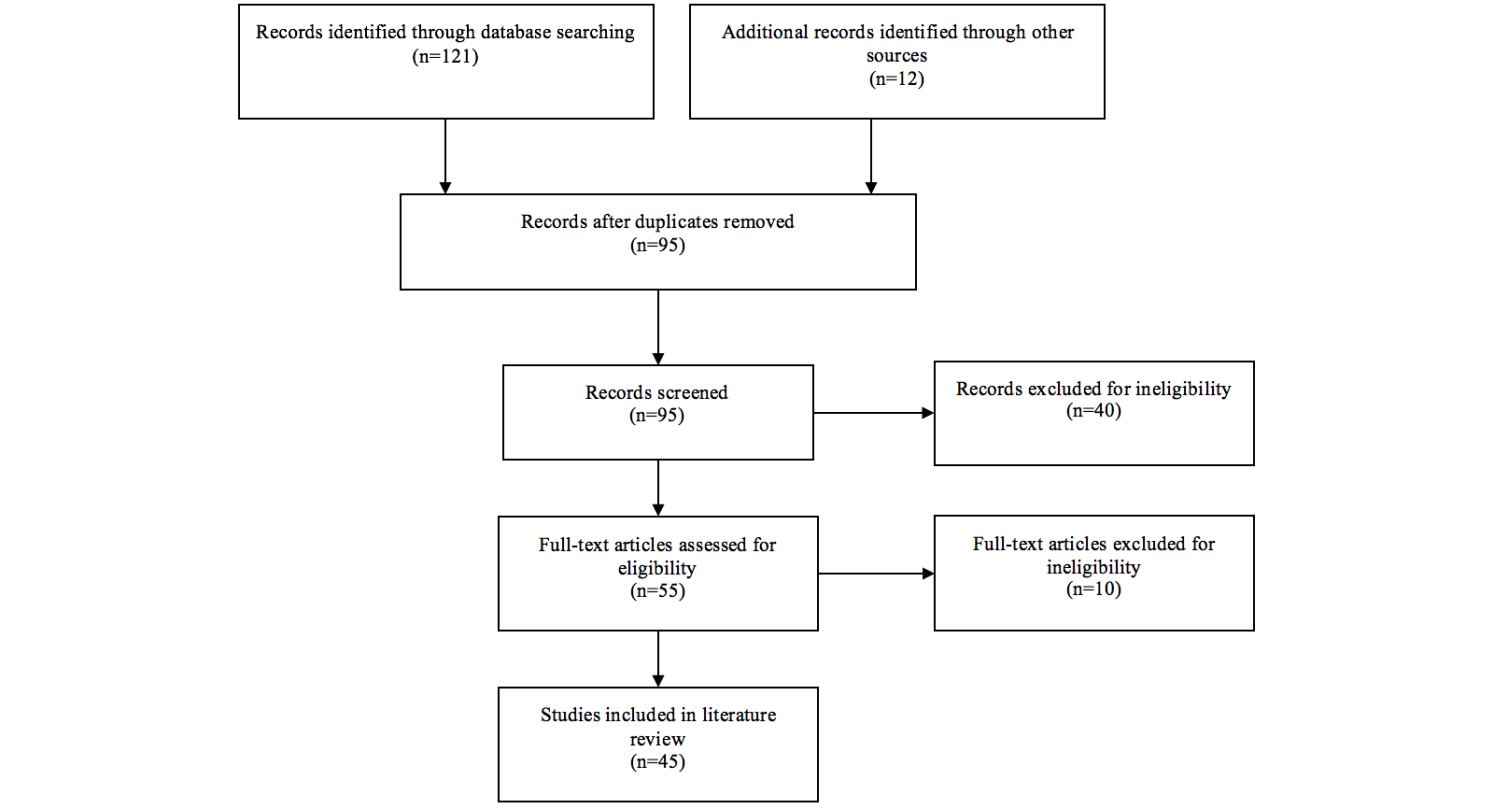
Flow diagram of search procedure.

The search protocol described above originally resulted in 121 references being returned through the database searches and 12 additional references through other sources or hand searching. Duplicate articles were removed, resulting in a figure of 95. Subsequently, articles were removed having been reviewed against the inclusion and exclusion criteria, based on their title (19) or abstract (21). This resulted in 55 articles being obtained as full-text documents. Having read all these articles, a further 10 were excluded due to not meeting the inclusion and exclusion criteria; either because the studies did not actually involve people with dementia or because a touchscreen interface was not featured. In total, 45 articles were included for the final review. Figure 1 presents the flow diagram of the search procedure (adapted from [16]).

## Results

### Overview of Results

Forty-five articles met the inclusion criteria and were included for this review. Multimedia Appendix 1 presents the summarized results of the review, and information from these articles has been collated to provide an overview on this topic, organized according to the questions outlined in Textbox 1.

### Contexts of Use

A total of 3 broad categories of touchscreen technology utilization were identified during the review: (1) assessment and screening (14 articles); (2) assistive technology and cognitive rehabilitation (24 articles); and (3) leisure activities (9 articles). Two papers contained information pertaining to both an assistive device and a leisure activity and were counted in both categories. Multiple papers within both the assistive and leisure categories described the same devices or software, which is highlighted. Each of these categories have been discussed in detail. It is worth noting that the majority of papers in the “assessment and screening” category mostly describe the touchscreen device as a piece of equipment used to deliver a test, and rarely discuss the impact of selecting the specific technology.

#### Assessment and Screening

The first reported use of touchscreen technology with people with dementia was in 1986 [17], where the use of a touch-sensitive screen was compared with a conventional computer monitor with a peripheral response device to deliver 2 cognitive assessments or screening tests. In the early 1990s, 2 articles described the incorporation of touchscreen technology into cognitive assessments: the Cambridge Neuropsychological Test Automated Battery (CANTAB) [18] and the French-language Examen Cognitif par Ordinateur (ECO) [19]. Touchscreens have continued to be used for these purposes, evidenced by more recent examples delivering tests of global cognition [20] or batteries of cognitive tests [21-23] for the detection of dementia or mild cognitive impairment (MCI).

In addition to global cognitive assessment, several articles reported the use of touchscreen technology to deliver tests of specific cognitive functions: visual attention [24], working memory [25], executive functioning [26], and visuomotor skills [27,28]. The remaining article in this theme [29] used computerized maze tests presented on a touchscreen computer to predict driving performance.

The vast majority of these articles developed original tests for the touchscreen format such as the Edinburgh Dementia App [23] and the Touch Panel-type Dementia Assessment Scale [22]. Only one study reported the adaptation of an existing test; the sparse-letter display test [24], which had previously been presented on a computer but not using the touchscreen format.

#### Assistive Technology and Cognitive Rehabilitation

The majority of articles describe the use of touchscreen technology to provide an assistive function for the person with dementia or their caregivers, or to present interactive cognitive exercises.

Five of the reviewed papers discussed the Computer Interactive Reminiscence and Conversation Aid (CIRCA), a communication support tool using digital reminiscence materials to stimulate conversation between the person with dementia and a conversation partner [30-34]. Several other studies also used reminiscence materials presented on a touchscreen interface to provide other assistive functions [9,35-39]. The use of touchscreen technology to support therapists was also evident in the context of art therapy and occupational therapy [40-42]. Several articles reported the use of touchscreen technology to address multiple activities of daily living (ADL) for people with dementia [43-46], including calendars, diaries, video calling, and location tracking. Although different terminology was used to describe their focus, the remaining articles categorized in this section used touchscreen technology to present cognitive exercises to people with dementia, either using originally designed software [47-51] or existing software [52].

#### Leisure Activities

Several of the aforementioned articles have featured games or leisure activities; however, these have been designed to assess cognition [21,26], provide cognitive stimulation [37,45], or to assist in the delivery of therapeutic interventions [40,41]. Very few studies focused on games or activities purely for entertainment or leisure purposes.

Three of the reviewed articles described “Living In the Moment” (LIM) [31,53,54], a suite of touchscreen games and activities that at various stages of the project included virtual environments, skill games, games of chance, and creative activities, the common factor being that they were all designed in partnership with people with dementia. Original design was also utilized in 3 articles; 2 focusing on musical creativity [55,56] and 1 to provide enjoyable activity either independently or in a group setting [39]. The remaining articles included in this section investigated the use of existing touchscreen activities, rather than those developed specifically for people with dementia [5,10,13].

### Touchscreen Technology Selection

Many, although not all, reviewed articles reported why they had chosen touchscreen technology. The reasons can be summarized into the following categories: the intuitive control method (9 articles), practicalities of administration (12 articles), the ability to customize and adapt (4 articles), and the multifunctional nature of the devices (10 articles). These reasons are explored further.

#### Intuitive Control

The touchscreen control method is widely regarded as intuitive [5,10,17,47] and easy to use [25,39], making it highly advantageous for people with dementia. Eliminating the need for external input devices, for example, a keyboard and a mouse, is beneficial as it reduces the cognitive load required to input information [10,17,24,47]. This was addressed directly in Tippett & Sergio [28], where the performance of people with dementia on a visuomotor test was highest when the touch-sensitive interface was placed directly over the computer monitor as opposed to when placed in front or to the side. A similar method was used in the study by Carr et al [17], who reported that participants in the group using an external response board would sometimes intuitively reach out to touch the screen. An alternative example can be seen in Ott et al [29], where participants were required to use a stylus to trace a path through the maze in order to replicate the “natural” method of using a paper and pen.

#### Practicalities

In administering cognitive tests, touchscreen computers are seen as a more practical solution for a number of reasons. These include increased accuracy of data input [18,25,29], flexible but also standardized administration [25], reduction in administration bias by avoiding experimenter effects [20], financially efficient implementation [22,25,29], and the wide availability of this technology in health care settings [23].

In addition, the use of touchscreen computers reduces the practical requirement for members of staff to prepare and manage multiple materials, for example, reminiscence materials [30,33,38,42,52]. This is highlighted as a potential time-saving measure for often busy clinical staff [41].

#### Customization

Programs and apps presented on touchscreen devices can be designed to facilitate customization, which allows for easy adaptation and consequently they can be responsive to the needs of the users [13,25,37,40,41]. Presenting customization options within programs in an accessible format allows a caregiver or therapist to tailor the program to each individual [40,41]. This is particularly beneficial for people with dementia as programs can become responsive to change in their cognitive functioning and abilities over time. For example, with games, it is important to include difficulty options so that each player can find a suitable entry point [37]. Another benefit to customization highlighted in the literature is with regards to administering cognitive assessments, where being able to easily manipulate experimental parameters can allow for repeat testing while avoiding learned responses [25].

#### Multifunctional Use

A further advantage of touchscreen devices such as tablets and smartphones is that they can provide a wide range of functions for the user. As is reflected in the literature, these devices can address the multiple needs of people with dementia, for example, increasing socialization, providing memory prompts, facilitating activities, and delivering educative tools [10,13,36,37,44]. During reminiscence activities, for example, photographs and music can be accessed simultaneously, increasing their potential to trigger memories [38]. The fact that a wide variety of downloadable apps can be added to such devices only increases the availability of these functions [5,52]. It is also reported that built-in and attachable accessories, for example, cameras [35] and sensors [48] can even further increase the functionality available through these devices.

### Hardware and Software

Where reported in the literature, information related to the hardware and software used in the reviewed studies is discussed here. The information that was judged as most relevant was screen size and the model of tablet devices or smartphones and their operating system (OS). To allow for easier comparison, all screen sizes have been converted into inches (diagonal), if not already presented in this unit.

#### Screen Size

The touchscreen devices used in the reviewed articles range in size, largely determined by whether a monitor (largest), tablet, or smartphone (smallest) was used. Fourteen articles reported and specified using a touchscreen monitor or a touch-sensitive interface in combination with a monitor [17,21-25,28-30,33,34, 40, 46,51,53]. Screen size in these studies ranged from 14˝ to 32˝ with a mode size of 20˝. Six articles reported and specified using a tablet device, all with a screen size of 9.7˝ [5,10,39,42,52]. Three articles reported and specified using a mobile smartphone, with sizes of 2.8˝ [46], 3.5˝ [13], and 3.8˝ [43].

With regard to size, a larger screen can be advantageous for people with cognitive impairment, particularly when there is the addition of a visual impairment [56]. This would support the use of monitors, however the portability of tablet devices and smartphones is also seen as advantageous [25], as is the availability and ease of access to downloadable apps [5,52]. There should be consideration for the suitable placement of tablet devices during interactions, given their size and weight, with the recommendation of placing the device on a surface (eg, table) and raising the height to a comfortable level for the user to reduce muscle stress [25]. Finally, the small size of smartphone screens has been highlighted as a potential issue for people with dementia during user testing [43].

#### Models and Operating System

All the studies that reported using tablets, and specified which device, used an Apple iPad [5,10,39,42,52]. In discussing the reason for selecting an iPad, and therefore the Apple iOS, Lim et al [10] commented on its ease of use when compared with Android OS or Windows OS, a factor that is particularly important where the intended users are people with dementia. Android [48], Windows [43] and Apple [13] were each used as the OS in studies that specified smartphone use. In the study by Zmily et al [48] involving the use of near-field communication (NFC) technology, the Android OS was selected primarily because, at the time, the majority of mobile devices with NFC functionality used Android. Commenting on app development, Pang and Kwong [37] stated that apps designed for people with dementia should be developed for both Apple and Android to allow people the choice in what device to purchase, particularly in relation to cost.

### Independent Use

The use of touchscreen technology in the reviewed articles involved a range of interaction levels between the people with dementia and the devices. Supported use was common, that is, where the person with dementia interacts with the technology in the presence of a clinician or carer, where input may be encouraged or shared [23,28,30,33,34,38,41,42,56]. Many studies involved devices that were designed for independent use or used existing devices that were utilized independently by the person with dementia [9,10,13, 20,22,24,26,32,35, 37,43-45,47,53,54]. In some cases, independent use was successful. For example, Lim et al [10] reported that half their participants were able to use an iPad independently for leisure activities, and a quarter were able to store and charge the device without support. Participants using the LIM games were left alone to interact with the touchscreen and the majority were able to navigate the system independently, even at the prototype stage [53]. Two thirds of participants were able to use the Companion system independently, although the remaining third were not, with the authors citing personal motivation and physical impairment as potential factors [9]. Although the “COGKNOW” system was designed for independent use by people with dementia, in practice it was found that those people who lived with a partner tended to rely on them for support [44]. Several articles reported positive factors for people with dementia associated with independent use of the touchscreen devices, including relaxation [9], enjoyment [9,45,54], autonomy [9,45,54], motivation [26], socialization [32], and engagement [54].

In reviewing the articles for evidence of independent touchscreen use, key factors emerge relating to the potential for successful outcomes; namely, training, use of prompts, integrated feedback, and visual design. Each of these factors will now be discussed.

#### Training

There were many examples of studies using a training or demonstration phase before participants were expected to use a device independently [13,24-26,28,48,57]. In several cases, this involved the researcher or clinician demonstrating or instructing device use, followed by a familiarization phase where the participant would be observed using the device so that their understanding could be verified [24,25,28,57]. In one example using this method, the familiarization phase would only end once the clinician was satisfied that the participant could use the device independently, up to a maximum of 8 trials [28]. In another example, a simplified version of the actual trial test was used during this phase to prevent learning bias [24]. Zmily et al [48] predicted that this demonstration would be necessary, given that the target population is generally less experienced using computer devices, which was supported in their results. In their case study, Astell et al [13] concluded that the participant’s successful adoption of several forms of new technology was achieved because of the high level of appropriate training and support delivered by the researcher, which will not always be feasible.

#### Prompts

Many of the articles described the use of integrated prompts within their software to direct or regain the attention of the user, although the outcomes are varied. In developing the LIM games, the research team considered and experimented with many different forms of prompts including text boxes, animations, the spoken voice, and an avatar [53,54]. The idea of an avatar was rejected due to the potential for it to be overly distracting, while the spoken voice prompt was implemented but often ignored (possibly due to its synthetic nature being unrecognizable), or relied on too heavily, resulting in a passive experience where the user would just wait until they next received an instruction. In contrast, the text boxes and animations were found to be more successful, with the conclusion being that overly intrusive prompts were unnecessary [54]. Other studies reported using spoken prompts in their programs [20,22,35,48], either through human recording or synthesized text-to-speech. Inoue et al [22] reported that participants were more likely to find prompts useful in the earlier stages of dementia. In Meiland et al [44], the use of visual and audio prompts was reported to be largely unsuccessful, with users either not noticing the prompt or ignoring it.

There was also variety between the studies in how prompts were triggered, for example, following a period of inactivity [53,54]; following a predetermined number of errors [26]; or using artificial intelligence to detect a reduction in engagement, measured through eye-tracking and screen touches [41].

#### Feedback

The importance of feedback in response to user input when designing or selecting touchscreen software for use by people with dementia was discussed in several articles [24,54,56]. Feedback should involve either an animation or sound effect (or both) contextual to the input and should be immediate, to acknowledge the user interaction [54].

#### Visual Design

When designing interfaces specifically for people with dementia on touchscreen devices, the reviewed literature recommends the avoidance of complexity [35,37,40,56]. The number of steps to navigate or achieve goals should be kept to a minimum [35-37,56], with uncluttered interfaces [56], and the consistent use of colors and icons so that users have a sense of context [35-37]. The traditional design of apps may be problematic for people with dementia, with drop-down menus and ambiguous icons without text, and therefore should be avoided [36,37]. Icons, text, and graphics should be appropriately sized for people who may have visual impairment [36,37,47] and the interactive elements should be of a large enough size to allow for less precise motor control [47].

The multitouch control method popular on market-leading touchscreen devices has the potential to allow for easier and more engaging interactions for people with dementia [41]. However, with multitouch, there is the risk of accidental gestures caused by users resting their hand on one part of the screen while interacting with another [17,56], although considered programming can prevent this [17,41]. Using familiar imagery to cue users into their activity can be helpful for people with cognitive impairment [54], and offering activities that are familiar to people, such as virtual representations of everyday environments to explore [53] or digital versions of existing games to play [10] has also shown to be popular with this population.

To support the design process, Astell et al [33] recommended educating all members of the research and development team on dementia and enabling everyone to spend time talking with people with dementia and seeking their input. An iterative design process in collaboration with users is also recommended [32,53]. This can reduce the risk of releasing products that have poor performance, stability issues, or are not fit for purpose, which is highlighted as being crucial in order to achieve acceptance and adoption by people with dementia, their families, and services supporting them [44].

## Discussion

### Application of Knowledge

Although the use of touchscreen technology with people with dementia is in its infancy across the board, of the 3 main contexts (assessment, ADL, and leisure) highlighted in the results, the most apparent gap in the literature is in the application of these devices for leisure activities. Only 8 articles were returned from the literature search that could be categorized in this area, and within these only 6 projects are featured, as multiple articles focused on the same work. This is all the more unusual given that worldwide the most popular app category in the market leading app store for smartphones and tablets is games. There is no reason to believe that a diagnosis of dementia should alter people’s interests and hobbies. Moreover, one of the biggest challenges for people with dementia and those who care for them is finding ways to provide stimulating and meaningful activities for them to engage with.

Understanding why touchscreen technology has been used with this population in the past can help when making decisions as to how it might be used in the future. This is particularly pertinent, given the speed with which this technology evolves, and the availability of new design features both internally (software) and externally (hardware). Having reviewed the literature, clearly what has attracted researchers, clinicians, and designers working with people with dementia to touchscreen technology is the intuitive control method. While not entirely a new technology (Carr and colleagues were heralding its use 30 years ago [17]), its increase in availability, popularity and affordability in recent years has perhaps provided a new entrance into personal computing for people with dementia. The practicalities, customization and multifunctional abilities discussed in the literature could to a certain extent also be applied to non-touchscreen computing devices. However, in combination with the intuitive control method, it is no surprise that this technology is gaining the interest of those working with people with dementia. Areas that might require further consideration include how customization can best be implemented to improve the accessibility of this technology and how, with such large numbers of apps available, to identify which ones might be suitable for people with dementia.

Perhaps the most difficult outcome to analyze relates to the hardware, as there is a potential disparity between what is most available and popular on the market (and therefore presents the most opportunity) and what might be the most appropriate for this population. The majority of studies featured in this review used larger touchscreen devices (20˝ being the most common). In comparison with the Apple iPad, which was the single most used device in the remaining studies, this is almost 4 times the size. It is likely that in some of these cases there was no choice to be made as tablet devices with “acceptable” hardware have only been widely available since 2010 [58]. Given the knowledge gained on software design, a larger sized interface would certainly be beneficial for this population. However, with tablet devices like the iPad offering so many easily accessible, low-cost applications, and their smaller size (comparatively) offering more portability, there are advantages to this technology too. There is perhaps not enough information currently to definitively answer this question, and it is unlikely that there will be a “one-size-fits-all” solution, given the variety of contexts and individual variations (eg, individual or group activity, age, presence of physical impairment). If the principles of interaction derived from the earlier studies featuring larger touchscreens could be achieved with tablets, then this might provide an accessible, economically viable approach going forward. It would also be sensible to consider the specific target population and context in advance of each study and consult with people with dementia and people in a caregiving role before making a decision.

### Limitations

It became apparent during the review that many articles did not report all the information that might be considered pertinent to the completion of a comprehensive overview of this topic. This lack, combined with the relatively modest number of articles identified, is a limiting factor in applying the findings. For example, if the studies that reported trials of apps or devices consistently included information about the age and severity of cognitive impairment experienced by people with dementia, this would advance the knowledge about how the technology could be used at various stages of the condition. This is not to assume that there would necessarily be a correlation, for as Kerssens et al [9] reported, independent use was related more to personal motivation or curiosity for the technology than the level of cognitive function.

Another potential limitation is that the review may not have uncovered all studies that involved the use of touchscreen technology with people with dementia. The decision was made to include only articles that directly referred to the use of a “touchscreen” (or “touch screen”) interface. Every effort was made to investigate alternative terminology but nothing consistent was found, therefore the presence of the term “touchscreen” (or “touch screen”) dictated the search results. It also highlights the small amount of direct research touchscreens have received with this population beyond being an alternative to pen-and-paper cognitive tests.

### Conclusions

The reviewed literature can be seen as an emerging body of evidence that people who have dementia can independently use touchscreen technology. Certainly, there are caveats here involving the appropriate level of support needed, both on a human and on a technological level, but there is clearly enough reason to warrant continued research in this area. The results have highlighted numerous learning outcomes while also identifying areas that are currently under-researched. It is clear that touchscreen devices are not only usable by people with dementia, but the wide array of functions available offer great potential to improve their lives in many different contexts.

## Multimedia Appendix 1

Summarized literature review results.

## Abbreviations

ADL: activities of daily living
LIM: Living In the Moment
NFC: near-field communication
OS: operating system
