# Supplementary material for: Studies Involving People With Dementia and Touchscreen Technology: A Literature Review
Source: JMIR Rehabil Assist Technol. 2016 Nov 4;3(2):e10. doi: 10.2196/rehab.5788 (PMC5454556; doi:10.2196/rehab.5788)
Supplement: Supplementary file 1 [file rehab_v3i2e10_app1.pdf]

Multimedia Appendix 1

Summarized literature review results

Publication Author	Purpose of technology	Reason for technology selection	Hardware/ software	Independent use	Cohort size (people living with dementia)	Participant age	Level of dementia
**Alm et al. 2004[a]**	Assistive technology	Fewer materials required	Personal computer with touchscreen monitor/interface	Supported use	6; 9	74.3 (57-95); 83 (65-95)	MMSE 15.6 (10-25); MMSE 16 (8-22)
Alm et al. 2007	Leisure activities	?	Personal computer with touchscreen monitor/interface	Independent use; automated prompts	5; 6	?	?
**Alm et al. 2009[a]**	Assistive technology & leisure activities	?	?	?	N/A	N/A	N/A
**Armstrong et al. 2010[a]**	Assistive technology	?	Touchscreen smartphone, Windows software	Independent use	N/A	N/A	N/A
Astell et al. 2009	Assistive technology	?	?	Independent use	N/A	N/A	N/A
Astell et al. 2010	Assistive technology	Fewer materials required	Personal computer with touchscreen monitor/interface	Supported use	11	83.5 (65-95)	MMSE 15.9 (9-23)

**Publication Author**	**Purpose of technology**	**Reason for technology selection**	**Hardware/ software**	**Independent use**	**Cohort size (people living with dementia)**	**Participant age**	**Level of dementia**
**Astell, Alm et al. 2014**[a]	Leisure activities	?	?	Independent use; automated prompts	100+	?	?
**Astell, Malone et al. 2014**[a]	Leisure activities	Customisation, multi-functional use	Touchscreen smartphone, Apple software	Independent use; training	1	63	?
Carr et al. 1986	Assessment/ screening	Intuitive control, reduce cognitive load	Personal computer with touchscreen monitor/interface	?	14	82.2	Unspecified test 8.1
Davies et al. 2009	Assistive technology	?	Personal computer with touchscreen monitor/interface, touchscreen smartphone	?	17	68.1 (56-86)	?
Fukui et al. 2015	Assessment/ screening	?	Personal computer with touchscreen monitor/interface	?	124	75.6	MMSE <26
González et al. 2013	Cognitive rehabilitation	Intuitive control, reduce cognitive load	?	Independent use	?	?	MMSE >23
Hoey et al. 2010	Assistive technology	Customisation	Personal computer with touchscreen monitor/interface	?	N/A	N/A	N/A

**Publication Author**	**Purpose of technology**	**Reason for technology selection**	**Hardware/ software**	**Independent use**	**Cohort size (people living with dementia)**	**Participant age**	**Level of dementia**
Hofmann et al. 2003	Cognitive rehabilitation	?	Personal computer with touchscreen monitor/interface	?	9	68.1	19.6
Hofmann, Hock, Kühler et al. 1996	Cognitive rehabilitation	?	?	?	10	69 (49-86)	MMSE 19.4 (12-23)
Hofmann, Hock & Müller-Spahn 1996	Cognitive rehabilitation	?	?	?	4	50-85	MMSE 12-23
**Inoue et al. 2011**[a]	Assessment/ screening	Financially efficient	Personal computer with touchscreen monitor/interface	Independent use; automated prompts	34	79.2	21.8
Ishiwata et al. 2014	Assessment/ screening	Reduce administrator bias	?	Independent use; automated prompts	188	?	?
Kerssens et al. 2015	Assistive technology	?	?	Independent use	7	77 (60-88)	11-27
Kikhia et al. 2015	Assistive technology	Multi-functional use	?	Independent use; automated prompts	N/A	N/A	N/A
Kong 2015	Cognitive rehabilitation	Fewer materials required, multi-functional use	Touchscreen tablet computer, iOS software	?	10	64-84	MoCA 15.7

Publication Author	Purpose of technology	Reason for technology selection	Hardware/ software	Independent use	Cohort size (people living with dementia)	Participant age	Level of dementia
Leng et al. 2014	Leisure activities	Intuitive control, multi-functional use	Touchscreen tablet computer, iOS software	?	6	77 (68-86)	MMSE 21 (16-25)
Leuty et al. 2012	Assistive technology	Fewer materials required, customisation	?	Supported use	6	89.2	MMSE 16.5 (15-25)
Lim et al. 2013	Leisure activities	Intuitive control, reduce cognitive load, multi-functional use	Touchscreen tablet computer, iOS software	Independent use	21	73.5 (50-91)	Reisberg's Scale 2-5 ('Early-stage')
Manera et al. 2015	Assessment/ screening	?	?	Independent use; training	12	80.3 (70-90)	MMSE 18.4 (15-24)
Meiland et al. 2012	Assistive technology	Multi-functional use	?	Independent use; automated prompts	16; 14; 12	56-78; 57-90; 57-84	MMSE 17-25
**Nezerwa et al. 2014**[a]	Assistive technology	Multi-functional use	?	?	N/A	N/A	N/A
Nijhof et al. 2013	Assistive technology	?	?	Independent use	16	78 (58-86)	MMSE 22 (13-29)
Ott et al. 2008	Assessment/ screening	Increased accuracy of data input, financially efficient	Personal computer with touchscreen monitor/interface	?	88	75.8	MMSE 24

**Publication Author**	**Purpose of technology**	**Reason for technology selection**	**Hardware/ software**	**Independent use**	**Cohort size (people living with dementia)**	**Participant age**	**Level of dementia**
Pang et al. 2015	Assistive technology	Customisation, multi-functional use	?	Independent use	N/A	N/A	N/A
Pignatti et al. 2005	Assessment/ screening	Reduce cognitive load	Personal computer with touchscreen monitor/interface	Independent use; training	16	74.8 (62-83)	MMSE 21.9 (15-27)
Pringle et al. 2013	Assistive technology	Fewer materials required, multi-functional use	?	Supported use	8	?	?
Purves et al. 2014	Assistive technology	?	Personal computer with touchscreen monitor/interface	Supported use	3	81-90	'Moderate' (anecdotal)
Riley 2007	Leisure activities	?	?	?	N/A	N/A	N/A
Riley, et al. 2009	Leisure activities	?	?	Supported use	10; 10	?	'Mild-Severe' (anecdotal); 'Mild-Moderate' (anecdotal)
**Ritchie et al. 1993**[a]	Assessment/ screening	?	?	?	N/A	N/A	N/A
**Sahakian & Owen 1992**[a]	Assessment/ screening	Increased accuracy of data input	?	Independent use; training	N/A	N/A	N/A
**Sahakian et al. 1993**[a]	Assessment/ screening	?	?	?	65	66.7 (52-84)	MMSE 17.3 (8-28)

Publication Author	Purpose of technology	Reason for technology selection	Hardware/ software	Independent use	Cohort size (people living with dementia)	Participant age	Level of dementia
Satler et al. 2015	Assessment/ screening	Ease of use, increased accuracy of data input, standardized administration, financially efficient, customisation	Personal computer with touchscreen monitor/interface	Independent use; training	22	78.3	?
Tippett et al. 2006	Assessment/ screening	?	Personal computer with touchscreen monitor/interface	Independent use; training	14	79.7	MMSE 12-28
Tomori et al. 2015	Assistive technology	Fewer materials required	Touchscreen tablet computer, iOS software	Supported use	116	78.5	MMSE 16.6
Verheij et al. 2012	Assessment/ screening	?	?	?	16	75.4 (66-88)	MMSE 24.1 (19-28)
Weir et al. 2014	Assessment/ screening	Availability	Personal computer with touchscreen monitor/interface	Supported use	N/A	N/A	N/A
**Yamagata et al. 2013**[a]	Assistive technology	Ease of use	Touchscreen tablet computer, iOS software	?	?	?	?
Zmily et al. 2014	Cognitive rehabilitation	Multi-functional use	Android software	Independent use; automated prompts	10	75	'Early-stage' (not defined)

[a]*Indicates an article that was not identified through database searching*
